# Bonding Agents in Pit and Fissure Sealants: A Review

**DOI:** 10.5005/jp-journals-10005-1011

**Published:** 2009-12-26

**Authors:** Usha Mohan Das, Suma G

**Affiliations:** 1 Principal, Professor and Head, Department of Pedodontics and Preventive Dentistry, Bapuji Dental College and Hospital Davangere-577004, Karnataka, India; 2 Senior Lecturer, Department of Pedodontics and Preventive Dentistry, VS Dental College and Hospital, Bengaluru Karnataka, India

**Keywords:** Bonding agents, pit and fissure sealants, sealant evaluation.

## Abstract

Dental adhesive systems used for bonding dental resins to enamel and dentin have evolved through several "generations," with changes in chemistry, mechanism, number of bottles, application technique, and clinical effectiveness. The trend in the latest generation of dental bonding systems is to reduce the number of components and clinical placement steps. The introduction of i bond, a single-bottle dental adhesive system, is the latest of the new generation materials, and combines etchant, adhesive, and desensitizer in one component. This paper describes different dentin bonding agents, its evolution, mechanism of action and different commercially available dentin bonding agents and their role in the retention of pit and fissure sealant.

## INTRODUCTION

Dentin/enamel adhesives allow bonding of resin-based composites and compomers to primary and permanent teeth. Adhesives have been developed with reported dentin bond strengths exceeding that of enamel.^1^

The preventive function of pit and fissure sealant is achieved by the adherence of the material to the acid etched enamel surface thus physically occluding the pits and fissure from the rest of the oral environment. The clinical success of adhesives allows for more conservative preparation when using composite restorative materials.

The use of the bonding agent may alter the rheology of the material allowing it to flow better into the fissures and acid etched surface. The use of phosphate containing bonding agent may result in chemical bonding to the calcium of the enamel surface as well as micromechanical retention from the sealant.^2^

Dentin bonding systems consist of bifunctional molecules: (1) A methacrylate group that bonds to the restorative resin by chemical interaction and (2) a functional group that is able to penetrate wet dentin surface. The use of dentin bonding agents between the tooth and fissure sealant can be beneficial for reducing microleakage when there is contamination of the enamel.^3^

Retention rates are of interest as sealant effectiveness is directly related to its retention and completely sealed fissures should not develop caries. Studies have shown improved results when an intermediate bonding layer is applied between enamel and sealant which increases, bond strength, reduce microleakage and enhances flow of resins into fissures.

## CLASSIFICATION OF BONDING AGENTS

*First generation bonding agents:* The foundation of modern adhesive dentistry was laid in 1995, Buonocore discovered that acrylic resin could be bonded to human enamel that was conditioned with 85% phosphoric acid for 30 seconds. Buonocore found that acid-etching enamel caused a large increase in resin enamel bond strength (ca. 20-25 MPa). But they tried those same procedures on dentin, they were disappointed to find that the resin-dentin bond strengths to acid-etched dentin was very low (5-10 MPa). He predicted that this bonding technique could be used in various dental procedures, including class III and class V restorations and pit and fissure sealants. They included NPG-GMA.

(*N*-phenylglycine glycidyl methacrylate), the polyurethanes, and the cyanoacrylates. SS White’s Cervident which had very poor clinical results when used to restore cervical erosion lesions without mechanical retention.

*A second generation bonding agents:* These dentin bonding agents was introduced in clinical use in the early 1980’s. Some of them are dual cure scotchbond, 3M ESPE, Bondlite, Kerr, they are no longer widely used. Most were halophosphorous esters of unfilled resin such as Bis GMA (Bisphenol a glycidyl methacrylate) or HEMA (Hydroxy ethyl methacrylate. They bonded to dentin via surface wetting and interaction between their phosphate groups and calcium ion in the smear layer. Shear dentin bond strengths were only about 1-10 Mpa. A major problem in clinical performance of these agents is that they bonded to the smear layer rather than to the dentin itself. Thus their bond strength was limited by the cohesive strength of the smear layer or by the weak, unstable adhesion of smear layer to the underlying dentin. There were three types of secondgeneration products.

1. Etched tubule dentin bonding agents: Representative brand: Dentin bonding system (Den-Mat).
2. Phosphate ester dentin bonding agents: Representative brands: Bondlite (SDS/Kerr), Creation bond (Den-Mat), Prisma universal bond (Caulk), and Scotchbond (3M).
3. Polyurethane dentin bonding agents: Representative brand: Dentin-Adhesit (Ivoclar Vivadent).

*A third generation* of bonding agents was introduced in the mid to late 1980’s and these either modified or removed the smear layer to permit resin penetration in to the underlying dentin.

Examples are: Scotchbond 2 (3M ESPE), Gluma (Bayer), Tenure (Den-mat corporation, Prisma universal bond 2 and 3 (Dentsply caulk), Syntac classic (Ivoclar Vivadent) and XR bonding system (Kerr)^4^ Composition:^4^

*Fourth generation* dentinal adhesive were developed in the early to mid 1990’s and represent a significant advancement in dentistry. They are based on the ‘total-etch’ concept were both enamel and dentin are simultaneously etched with 37% phosphoric acid for 15 seconds. Following this the tooth is washed and gently dried to leave the dentin surface moist so as to prevent the exposed collagen network. Next a primer of hydrophilic monomers is applied. Due to their volatile nature, the solvent displaces water from collagen network and allows penetration of the primers. Finally the adhesive resin is applied which may be Bis GMA, UDMA with TEGDMA and HEMA. The adhesive resin copolymerizes with the primed dentin and also composite resin applied over it.

Introduction of this total etch concept was based on the work of Fusayama and others in Japan. Bertolotti and Kanca proposed a technique that involved phosphoric acid – etching of dentin as well as enamel followed by the application of relatively hydrophilic resins. Products such as All- Bond 2 (Bisco, Inc), Optibond FL (Kerr), Perma Quick (Ultradent products) and Scotch bond multipurpose (3M ESPE).^5^

With the development of fourth generation dentin bonding agents, the concept of hybrid layer formation was put forth as the mechanism of bonding to dentin. This concept was proposed by Nobo Nakabayashi in 1982. This layer is formed by the inter diffusion of the low viscosity monomers into the exposed collagen network and the intertubular dentin to form a micromechanical bond with dentin. The hybrid layer is very strong and tough when properly formed, and affords enormous micromechanical retention for resin composites.^6^

Another major advance in dentin bonding technique that occurred in the early 1990s was the "wet" (or moist) bonding technique developed by Dr. John Kanca, who discovered that if one left some residual water in acid-etched dentin, bond strengths could be doubled.^7^

**Examples:** Scotchbond multipurpose, All bond 2.

**Composition:** Scotchbond multipurpose.^4^

All bond 2.^4^

*Fifth generation bonding agents:* The fifth-generation systems introduced during the mid 1990s combined the primer and adhesive while maintaining high bond strengths. It is simplified version of the fourth generation adhesive. In this primer and adhesive are present in the same bottle rather than two different bottles. Though they require fewer steps in achieve dentin bonding, these agents are inferior to fourth generation bonding agents in terms of their bond strength. Examples are single bond (3M), one-step (Bisco), Gluma comfort bond (Heraeus Kulzer), Opibond solo (kerr). The new self-etching primers can utilize the smear layer as a legitimate bonding substrate

When single bottle primer/adhesives are applied, the solvent may diffuse into the water, instead of vice versa forcing adhesive monomers to undergo phase changes, forming blisters, resin globules, etc.

A great advantage is that self etching primers are designed to be used on dry dentin. Although one should not over desiccate dentin, the dentin surface can be briefly dried following cavity preparation because the dentin is mineralized. This avoids all issues of how moist or wet the dentin should be prior to bonding. It is far easier to produce uniform dryness than it is uniform wetness. Another advantage of these self etching primer systems is that they do not etch very far into the dentin beneath smear layers. This avoids removal of smear plugs in the tubules and seems to be responsible for the lack of postoperative sensitivity associated with these technique-insensitive adhesive systems.^8^

Even though the hybrid layer is thin, resin dentin bond strengths are very high. The first marketed modern self etching primer (ca. 1989-1991) was Scotch Prep.

**Composition:** Single bond, one step, Gluma comfort bond.^4^

*Sixth generation bonding agents:* The sixth-generation dental adhesive systems introduced in the late 1990s and early 2000s eliminated the separate acid-etching step by incorporating an acidic primer that was placed on the enamel and dentin after tooth preparation and isolation from saliva. This dentinal adhesive, include self etching primer where primer and etchant are in one bottle and the adhesive resin is in another bottle. They show good bond strength to dentin but not to enamel. Examples are-Prompt-L-Pop (3M), Clearfil SE Bond (Kuraray) Xeno (Dentsply) It is important to note that although self-etching.

Primer products bond well to roughened or prepared enamel, they form a significantly weaker bond to unprepared enamel if the enamel is not first etched with a standard phosphoric acid etchant. The bond strength to dentin and enamel is lower than the fourth and fifth generation systems.

**Composition:** Clearfill SE bond.^4^

*Seventh generation bonding agents:* Seventh-generation systems were introduced in late 2002 and it combined etchant, primer, and adhesive in a single bottle, eliminating an additional mixing and/or placement step over the sixthgeneration systems. i-bond is a seventh-generation, singlecomponent, no-mix, one-step application dental adhesive with an etchant, adhesive, desensitizer, and photoinitiator. This i-bond (Heraeus kulzer) was said to perform etching, disinfecting, priming and bonding in a single step. The benefits using this bonding agent increases patient comfort, reduces chairside time, decreases contamination, increases efficacy, which would promising in pediatric patients in preventing pit and fissure caries. It contains UDMA, 4- META in an acetone/water solvent and Gluma desensitizer, 4-META monomer is responsible for conditioning due to its acid function. UDMA is used as cross linking and film forming monomer to add the required stability to the adhesive layer.^9^

**Examples:** G-bond- GC corp, i-bond- Heraeus Kulzer.

**Composition;**^10^

## CLINICAL TRAILS

A modification of the sealant application technique was proposed by Hitt and Feigal (1992), with the use of a dentin bonding agent layer between the etched enamel and the sealant. Some studies confirmed the benefits of the application of bonding agents under sealants in etched enamel that was contaminated by saliva (Choe et al, 1997: Fritz et al, 1998). Such studies showed reduction of microleakage and increase in the retention rate of the sealant. A low-viscosity hydrophilic material bonding layer, as part of or under the actual sealant, is recommended for longterm retention and effectiveness. Feigal, Hitt and Splieth (1993) believe that using bonding agent is useful to increase fissure sealant retention on teeth contaminated with saliva.^11^

The results of the clinical study by Boksman et al (1993), reported that the use of bonding does not increase retention.^12^ Complete penetration of the etchant into the pits and fissures is essential for sealant retention. However, there have been reports of insufficient penetration of the phosphoric acid etchant into the fissure system.^13^

Symons et al (1996) reported that sealant penetration of enamel fissures was enhanced by the use of the All- bond 2 (Bisco) and Scotchbond multipurpose (3M dental) systems.^14^ Swift et al in 1998 studied and suggested that water based primer or adhesives like Scotch bond multipurpose may be less effective than acetone or ethanol based adhesive systems like Tenure primer. *In vitro* and *in vivo* studies report that use of a bonding agent will improve the bond strength and minimize microleakage.^11^ Over the last decades, the application of bonding agent underneath the sealant has been widely suggested to improve adhesion to acid etched enamel Grande et al in (2000) compared the retention of multiuse bonding agents (Optibond) with that of conventional sealant used as pit and fissure sealant and reported a better clinical performance with optibond.^15^

In another study, Feigal and Hebling in (2000) assessed the effect of bonding as an intermediate layer on microleakage reduction on saliva contaminated enamel and they also showed that using bonding agent as an intermediate layer reduces microleakage on saliva-contaminated enamel.^11^

The use of a hydrophilic adhesive prior to sealant placement improves retention of the sealant and decreases microleakage, the use of self-etching adhesive systems may be a valid and promising alternative to acid etching with phosphoric acid. These adhesives have recently been introduced to simplify the bonding procedures and reduce the technique sensitivity by eliminating the etching, rinsing and drying steps of the bonding protocol.^16^

Pashley et al in (2001) found no correlation between the bonds to enamel of these adhesive systems and their pH (weak, moderate or strong). So far, the literature does not provide a straight forward answer whether mild self-etch adhesives bonded to enamel can withstand the mechanical and chemical challenges of the oral cavity.^17^

The dental literature supports the use of tooth bonding adhesives when used according to the manufacturer’s instruction unique for each product, as being effective in primary and permanent teeth in enhancing retention of restorations, minimizing microleakage, and reducing sensitivity.^18^

In another research, Pinar et al (2004) assessed the clinical performance of sealants with and without a bonding agent and showed that the placement of bonding under the fissure sealant did not affect the clinical success of the sealant. It has been reported that the functional monomers in self-etching adhesives can chemically interact with hydroxyapatite^19^ which obviously contributes to the bonding effectiveness to enamel, despite the low etching aggressiveness of the primer. Other studies ( GM Correr et al, 2004) demonstrate that it is possible to use a dentin bonding agent, Opt i-bond, as pit and fissure sealant with good clinical results and acceptable performance under conditions of contamination.^11^

Venker D J et al (2004) reported that some clinical and laboratorial studies have shown that the association of selfetching adhesive systems to pit-and-fissure sealants is less effective than the use of etch-and-rinse adhesive systems.^20^

The addition of one- bottle system with primer and adhesive components together may enhance the initial bond to enamel and also improve the initial bond between the bonding agent and the sealant. The fifth generation single bottle system that were tested improved sealant success, showing significant hazard ratios of 0.53 for occlusal sealants and 0.35 for buccal/lingual sealants.^21^

A study by Arzu Pinar et al (2005) observed that the use of bonding agent as an intermediary layer between enamel and sealant did not affect sealant success during a 24-month period.^21^

Complete penetration of the etchant into the fissures should occur for a durable retention of the sealant to enamel. In a previous study by Bottenberg et al (1996) none of the tested commercially available etchants was able to penetrate farther than 17% of the total fissure depth in a fissure model.^22^ Therefore, the use of self-etching adhesive systems may be a valid and promising alternative to acid etching with phosphoric acid. These new self-etching adhesive systems are user-friendly by dental community and have been developed to simplify the bonding procedures and reduce the adhesive technique sensitivity since the enamel/ dentin acid etching, rinsing and drying steps are eliminated. Having less operative steps and a shorter chair time is particularly interesting when treating pediatric patients.^23^

The self-etching adhesive systems (Adper single bond and Clearfil S3-bond) used in the study Jaciara Miranda Gomes-Silva et al (2008) is considered as a weak selfetching primer (pH 2.5) and its hydrophilic acid functional monomer (10-MDP) has an intense chemical interaction with the hydroxyapatite. Clinically acceptable retention rates have been reported and may thus be the result of a two-fold mechanism: increased micromechanical retention in addition to the chemical interaction.^23^

Torres et al (2005) reported that the application of a bonding agent (Prime and Bond) layer underneath the resin pit-and-fissure sealant placement resulted in significantly higher Shear bond strength to the saliva-contaminated and noncontaminated groups. The authors also found that individual or simultaneous curing of the bonding agent and the sealant had no influence on bond strength to contaminated enamel. The different results found in the study by Jaciara Miranda et al (2008) may be attributed to the fact that adper single bond 2 etch-and-rinse adhesive system contains water as a co-solvent, which gives a lower volatility to this material compared to other adhesive systems that contain only acetone as a solvent.^23^

## DISCUSSION

In a study by Boksman L (1993) on 2 years clinical evaluation of retention of pit and fissures placed with and without use of bonding agents showed that, the use of Scotch bond 2 (third generation) and prisma universal bond (second generation) did not increase the long-term retention rate. At 6 months evaluation the sealant placed with use of bonding agent showed higher retention rate than its counterpart placed without a bonding agents. But at 2 years level the use of bonding agent prior to sealant application did not increase the retention rate.^13^

Third-generation dentin bonding systems: These systems alter or remove the smear layer prior to bonding and producebond strengths ranging from 16 to 2612-14 MPa. Some of the products produce bond strengths approaching those formed to enamel. Clinical retention rates of 100% at 2 years have been reported. 24

One set of criteria used to distinguish fourth generation products from earlier ones has been: Ability to bond as strongly to dentin as to enamel, ability to bond strongly to moist dentin, and technique insensitivity. An additional criterion may be the ability to bond, many different types of substrates (e.g., enamel, dentin, porcelain, base and noble metals, amalgam). Being able to bond to these substrates, generally means that, the dentin bonding agents enhances the strength of the bond of composite resin to them. The ability of dentin bonding agents to bond resin to metal has been demonstrated and commented upon by several researchers.^25^

In a research conducted by RJ Feigal (2000) on improved sealant retention on bonding agents, showed that the fourth generation dentin bonding agents ,Tenure primer and Scotch bond multipurpose primer, was not significantly effective in sealant retention.^13^

Fifth-generation products are essentially distinguished by being one-step or one-bottle products.

In general, these products have limitations. Many require at least as much time to apply or even more time than threecomponent products and they lack many of the components necessary to perform multisubstrate bonding. It also appears important to apply multiple coats of these agents so that there is an adequately thick resin layer on top of the hybrid layer.^26^

A study on retention of pit and fissure sealant with or without bonding agents by Arzu Pinar et al (2005) shows that, the clinically acceptable marginal integrity rates for sealants with a bonding agents after 3,6,12,24 months were 93%, 93%, 83% and 79% respectively. Similar way marginal discoloration were studied and no color change was seen were 96%, 93%, 81% and 75% respectively. Anatomic form of sealant were showed that after 6 months it is about 98%, after 12 months 83%, and after 24 months it was 79%. He concluded that the use of bonding agents as an intermediary layer between enamel and sealant did not affect sealant success during 24 months period.^9^

In a study by Baca P, et al (2007) on retention of three fissure sealant and a dentin bonding agents in caries prevention showed that the marginal leakage and bond to enamel of Optibond Solo (fifth generation), a hydrophilic one-bottle filled dentin adhesive, is not affected by humid conditions. Indeed, an increase in bond strength to enamel was reported when bonds were performed under wet conditions. Optibond Solo can achieve similar retention to fissure sealants at 12 months but appears to be more sensitive to the application technique.^27^

A clinical study on pit and fissure sealants with and without a seventh generation bonding agents (2008) showed that there was no significant effective in retention of pit and fissure sealants when compared to the conventional phosphoric acid etching technique.^28^,^29^

## CONCLUSION

Based on the results observed in several studies, the use of bonding agent as an intermediary layer between enamel and sealant did not affect sealant success. In situations in which control of saliva and isolation is impossible the use of bonding for increasing the quality of fissure sealant therapy is useful. Studies on latest generation bonding agent i- bond bonding agent showed the retention rate of sealant showed the same retention rate of sealant without using the bonding agents. So the bonding agent i-bond was not effective in retention of pit and fissure sealant when compared to the conventional phosphoric acid etching technique.
